# Inhibition of Alk signaling promotes the induction of human salivary-gland-derived organoids

**DOI:** 10.1242/dmm.045054

**Published:** 2020-09-28

**Authors:** Shohei Yoshimoto, Junko Yoshizumi, Hiromasa Anzai, Koichiro Morishita, Kazuhiko Okamura, Akimitsu Hiraki, Shuichi Hashimoto

**Affiliations:** 1 Section of Pathology, Department of Morphological Biology, Division of Biomedical Sciences, Fukuoka Dental College, Fukuoka 814-0193, Japan; 2 Oral Medicine Research Center, Fukuoka Dental College, Fukuoka 814-0193, Japan; 3 Department of Oral and Maxillofacial Surgery, Division of Oral and Medical Management, Fukuoka Dental College, Fukuoka 814-0193, Japan; 4 Department of Morphological Biology, Division of Biomedical Sciences, Fukuoka Dental College, Fukuoka 814-0193, Japan

**Keywords:** Human salivary glands, Organoid, Sialadenitis, TNF-α

## Abstract

Hyposalivation and xerostomia are the cause of several morbidities, such as dental caries, painful mucositis, oral fungal infections, sialadenitis and dysphagia. For these reasons, preservation of normal saliva secretion is critical for the maintenance of functionally normal oral homeostasis and for keeping good health. Several strategies for restoring salivary gland function have been reported, from different points of view, based on the use of salivary-gland-derived epithelial stem/progenitor cells and tissue engineering approaches to induce organoids that mimic *in vivo* salivary glands. In this study, we clarified that inhibition of activin receptor-like kinase (Alk) signaling was essential for the induction of human salivary-gland-derived organoids, and demonstrated the usefulness of such organoids as an inflammatory disease model. In inflammatory conditions like sialadenitis, in general, pro-inflammatory cytokines such as tumor necrosis factor-α (TNF-α, also known as TNF) are upregulated, but their function is still unclear. In our established human salivary-gland-derived organoid culture system, we successfully induced organoid swelling by stimulation with carbachol, a non-selective cholinergic agonist, and forskolin, an activator of cystic fibrosis transmembrane conductance regulator (CFTR). Furthermore, we found that this organoid swelling was inhibited by TNF-α. From these results, we could clarify the inhibitory function of TNF-α on saliva secretion *in vitro*. Thus, our established human salivary-gland-derived organoids would be useful for *in vitro* analyses of the morphological and functional changes involved in salivary gland dysfunctions in several research fields, such as pathobiology, inflammation and regenerative medicine.

This article has an associated First Person interview with the first author of the paper.

## Abstract (precis)

**Summary:** Human salivary-gland-derived organoids can be used for *in vitro* analyses of the morphological and functional changes associated with salivary gland diseases and dysfunctions.

## INTRODUCTION

The human salivary glands are exocrine glands that consist of acini and ducts, and produce ∼500-600 ml of saliva per day (Dawes et al., 2015; Watanabe et al., 1995). In humans, the three pairs of major salivary glands and the many other minor salivary glands are classified as serous, mucous or seromucous (mixed) types depending on the proportions of serous and/or mucous cells in the acini. Saliva, which consists mainly of water and some other components such as electrolytes, mucus and several kinds of enzymes, is critical for preserving oral health and homeostasis. Under normal conditions, saliva is produced by the acinar cells and secreted from the orifice of the mucosal surface through the several portions of ductules and ducts. If some unfavorable factors disrupt these acinar cell functions or the path of saliva flow, hyposalivation occurs. Hyposalivation is known to be caused by aging, inflammation, autoimmune diseases, side effects of medication or off-target radiation in the treatment of head and neck tumors. Hyposalivation triggers the induction of xerostomia, then hyposalivation and xerostomia can cause several morbidities, including dental caries, painful mucositis, oral fungal infections, sialadenitis and dysphagia (Dawes et al., 2015). Sialadenitis is one of the main factors of hyposalivation and is caused by bacterial and viral infections or autoimmune diseases. In these inflammatory conditions, pro-inflammatory cytokines such as tumor necrosis factor-α (TNF-α, also known as TNF) are upregulated (Mei et al., 2015; Limaye et al., 2019). However, the mechanisms of inflammation-induced hyposalivation and its therapeutic targets are not fully understood in the human salivary glands. To clarify the mechanisms and pathogenesis of salivary gland dysfunctions, establishment of culture systems to produce organoids that mimic human salivary glands are desired for *in vitro* analyses of morphological and functional changes that take place in such dysfunctions.

Organoids are organizational structures of cell aggregates, made using three-dimensional *in vitro* culture technologies, showing the self-organization and similar organ functionality as the tissue of origin (Sato et al., 2009, 2011). Such culture systems have been established from cells of several organs, including the colon, small intestine, stomach, liver and kidneys. In general, organoids are divided into two classes, depending on the cell of origin: those made from pluripotent stem cells, and those made from tissue-derived stem/progenitor cells. Tissue-derived organoids have become useful disease models in several research fields, such as pathobiology, developmental biology, inflammation, regenerative and cancer medicine, and drug discovery. Recently, functional salivary gland organoids mimicking salivary gland development were prepared from mouse embryonic stem cells (Tanaka et al., 2018). However, the culture systems for production of human salivary-gland-derived organoids are still under development, although the establishment of these culture systems is desired in many research fields. In this context, we successfully established a culture system of human salivary-gland-derived organoids and clarified that inhibition of activin receptor-like kinase (Alk) signaling is necessary for organoid formation, then we revealed the usefulness of this culture system in assays to quantitatively evaluate the effects of TNF-α in an inflammatory condition. Namely, we successfully induced an organoid swelling in our established human salivary-gland-derived organoid culture system, revealing the effect of swelling on saliva secretion through stimulation with carbachol, a non-selective cholinergic agonist, and forskolin, an activator of cystic fibrosis transmembrane conductance regulator (CFTR). Then, we found that these organoid swellings were inhibited by TNF-α. From these results, we could clarify the inhibitory function of TNF-α on saliva secretion *in vitro*. These results in this report will be beneficial for future *in vitro* analyses of the morphological and functional changes that take place in salivary gland dysfunctions in several research fields, including pathobiology, inflammation and regenerative medicine.

## RESULTS

### Salivary gland organoid formation

**Fig. 1.**
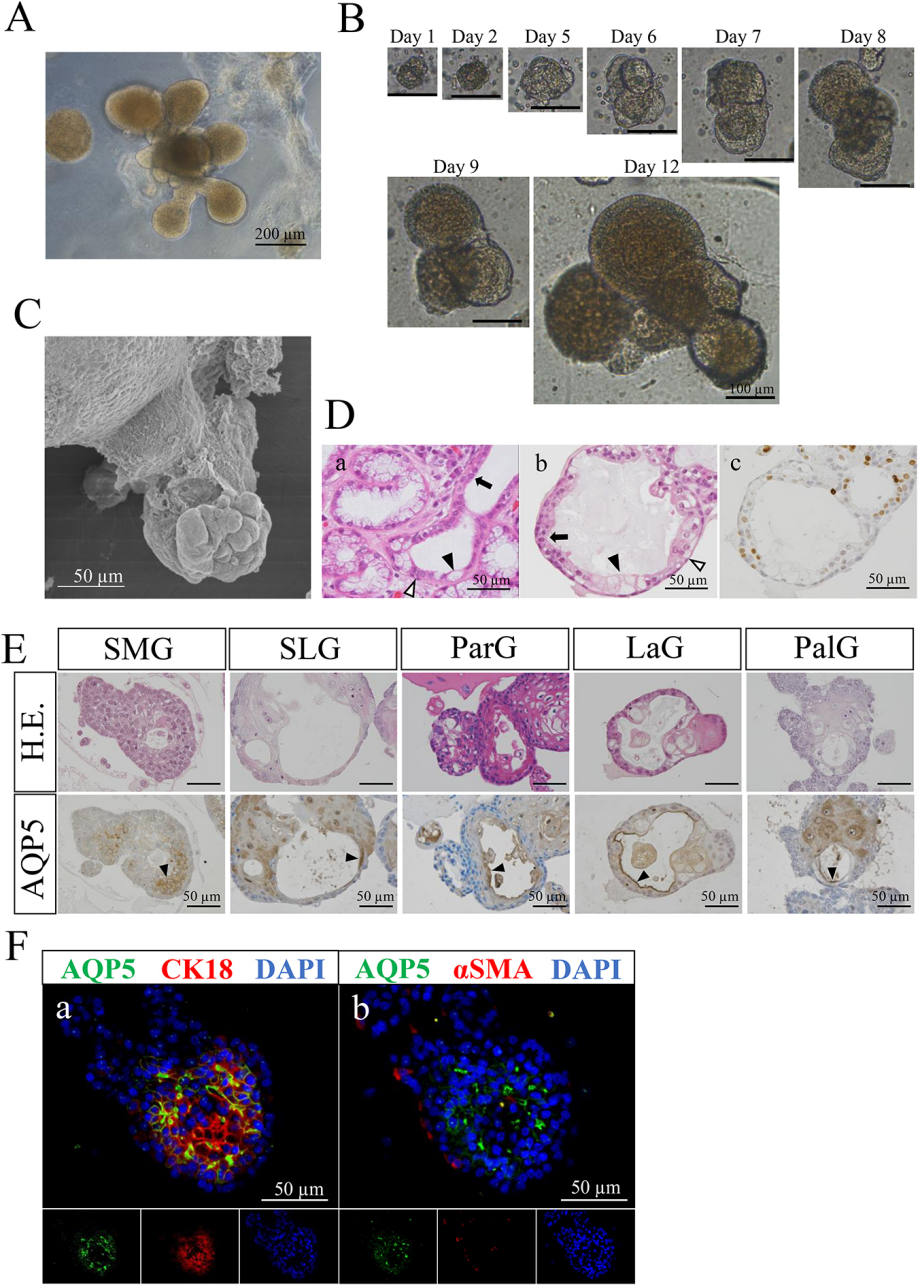
**Morphological and functional analyses of the human** **salivary-gland-derived organoids.** (A) Isolated human salivary-gland-derived cells were cultured in the solid gel phase of Matrigel™-GFR as a three-dimensional matrigel scaffold overlaid with CM/LS(+) for 16 days. Labial-gland-derived organoids are shown. Scale bar: 200 µm. (B) Timecourse of an isolated salivary-gland-derived cell growth. Images reveal clefts and budding formation of an organoid. Palatine-gland-derived organoids are shown. Scale bars: 100 µm. (C) Scanning electron micrograph of an organoid at day 10, showing an acinar-like surface architecture with clefts and buds. Image shows a labial-gland-derived organoid at day 10 of culture. Scale bar: 50 µm. (D) Comparison of normal human salivary gland (a) and organoid (b) tissues by H.E. staining. The organoid has two layers of cells, an inner lining of epithelial (arrow in b) and mucous cells (arrowhead in b), and an outer lining of cells (open arrowhead in b). These inner and outer layers are reminiscent of the luminal inner epithelial (arrow in a) and mucous (arrowhead in a) cells and outer myoepithelial cells (open arrowhead in a), respectively, in the region of the intercalated duct connecting to the secretory end piece of the normal salivary gland. Immunohistochemical staining of Ki-67 is shown in panel c. Organoid images show labial-gland-derived organoids at day 11. Scale bars: 50 µm. (E) Comparison of organoids derived from five different types of salivary glands (submandibular glands, SMG; sublingual glands, SLG; parotid glands, ParG; labial glands, LaG; palatine glands, PalG) by H.E. staining (upper panels) and aquaporin 5 (AQP5) immunohistochemical staining (lower panels). Positive staining of AQP5 is seen in the luminal side of organoids (arrowheads). Images show organoids at day 10 to 16. Scale bars: 50 µm. (F) Immunofluorescence staining of aquaporin 5 (AQP5; green, a and b), keratin 18 (CK18; red, a) and α-smooth muscle actin (α-SMA; red, b) is shown. Nuclei are stained with DAPI (blue, a and b). Individual fluorescence channel images are shown below the merge images. Submandibular-gland-derived organoids at day 16 are shown. Scale bars: 50 µm.

When the isolated cells from human salivary glands were cultured in complete medium (CM), spheroid-like organoids were formed within several days. However, these organoids only showed solid sphere formation with central keratinization, and no budding or branching was seen. In previous studies, activation of Alk signaling [which involves both bone morphogenetic protein (BMP) signaling and transforming growth factor-β (TGF-β) signaling] has been observed to cause salivary gland dysfunction (Yin et al., 2013, 2020; Sisto et al., 2020). On the other hand, Alk signaling inhibitors LDN193189 (an inhibitor of BMP receptors Alk2, Alk3 and Alk6, which are also known as ACVR1, BMPR1A and BMPR1B, respectively) and SB431542 (an inhibitor of TGF-β receptors Alk4, Alk5 and Alk7, which are also known as ACVR1B, TGFBR1 and ACVR1C, respectively) have been used in a process to form salivary glands from mouse pluripotent stem cells (Tanaka et al., 2018). Based on these reports, we applied the usage of Alk inhibitors LDN193189 (L) and SB431542 (S) for our methods to induce the formation of human salivary-gland-derived organoids. When the isolated cells were cultured in CM with L and S inhibitors [CM/LS(+)], organoids showed apparent morphological changes, including budding and/or branching features, after 16-17 days in culture (Fig. 1A). We investigated the process of organoid formation using a time-lapse daily image series (Fig. 1B). Some minor clefts were seen in the round organoid sphere at day 5. Organoids began to grow by budding and sprouting at culture day 6, and could be expanded for at least 1 month. Scanning electron microscope (SEM) observations identified the formation of acinar-like structures at the tip of the bud structures (Fig. 1C). When the normal human salivary gland (Fig. 1D, panel a) and organoid (Fig. 1D, panel b) tissues were compared by observing the histology of Hematoxylin-Eosin (H.E.) staining, the organoid revealed two layers of cells, an inner lining of epithelial (Fig. 1D, arrow in b) and mucous cells (Fig. 1D, arrowhead in b), and an outer lining of cells (Fig. 1D, open arrowhead in b). These inner and outer layers were reminiscent of the luminal inner epithelial (Fig. 1D, arrow in a) and mucous (Fig. 1D, arrowhead in a) cells, and the outer myoepithelial cells (Fig. 1D, open arrowhead in a) in the region of the intercalated duct connected to the secretory end piece of the normal salivary gland, respectively. Ki-67 (also known as MKI67)-positive proliferating cells were observed in labial-gland-derived organoids at day 11 (Fig. 1D, panel c) [Ki-67 labeling index (LI), 26.7%]. In CM/LS(+) culture conditions, organoids were successfully established from five different types of salivary glands (Fig. 1E). Predictably, parotid-gland-derived organoids consisted mainly of eosinophilic cells, suggesting the presence of serous cells. Conversely, the other salivary-gland-derived organoids contained mucous cells. Immunohistochemically, organoids showed a positive staining of aquaporin 5 (AQP5), an acinar cell marker, in the apical membrane of luminal cells in all organoids derived from all five different types of human salivary glands (Fig. 1E, arrowheads). Ki-67 immunohistochemical staining was also performed in organoids from the five different types of salivary glands, and the LI was calculated for each (Ki-67 LI: submandibular glands at day 16, 25.8%; sublingual glands at day 12, 14.1%; parotid glands at day 14, 15.9%; labial glands at day 11, 26.7%; palatine glands at day 10, 26.9%). Immunofluorescence revealed that organoids were positive for AQP5 on the cell-surface membrane of the inner cells (Fig. 1F; panel a, green). These inner cells were also positive for another acinar cell marker, keratin 18 (CK18, also known as KRT18) (Fig. 1F; panel a, red). Interestingly, α-smooth muscle actin (α-SMA, also known as ACTA2), a myoepithelial cell marker, was focally expressed in the outer peripheral cells (Fig. 1F; panel b, red), an expression pattern similar to that in the normal acini of human salivary glands. These findings show that the organoids display morphogenesis and protein expression similar to that of human salivary glands.

### Effects of Alk inhibition on the formation of human salivary-gland-derived organoids

**Fig. 2.**
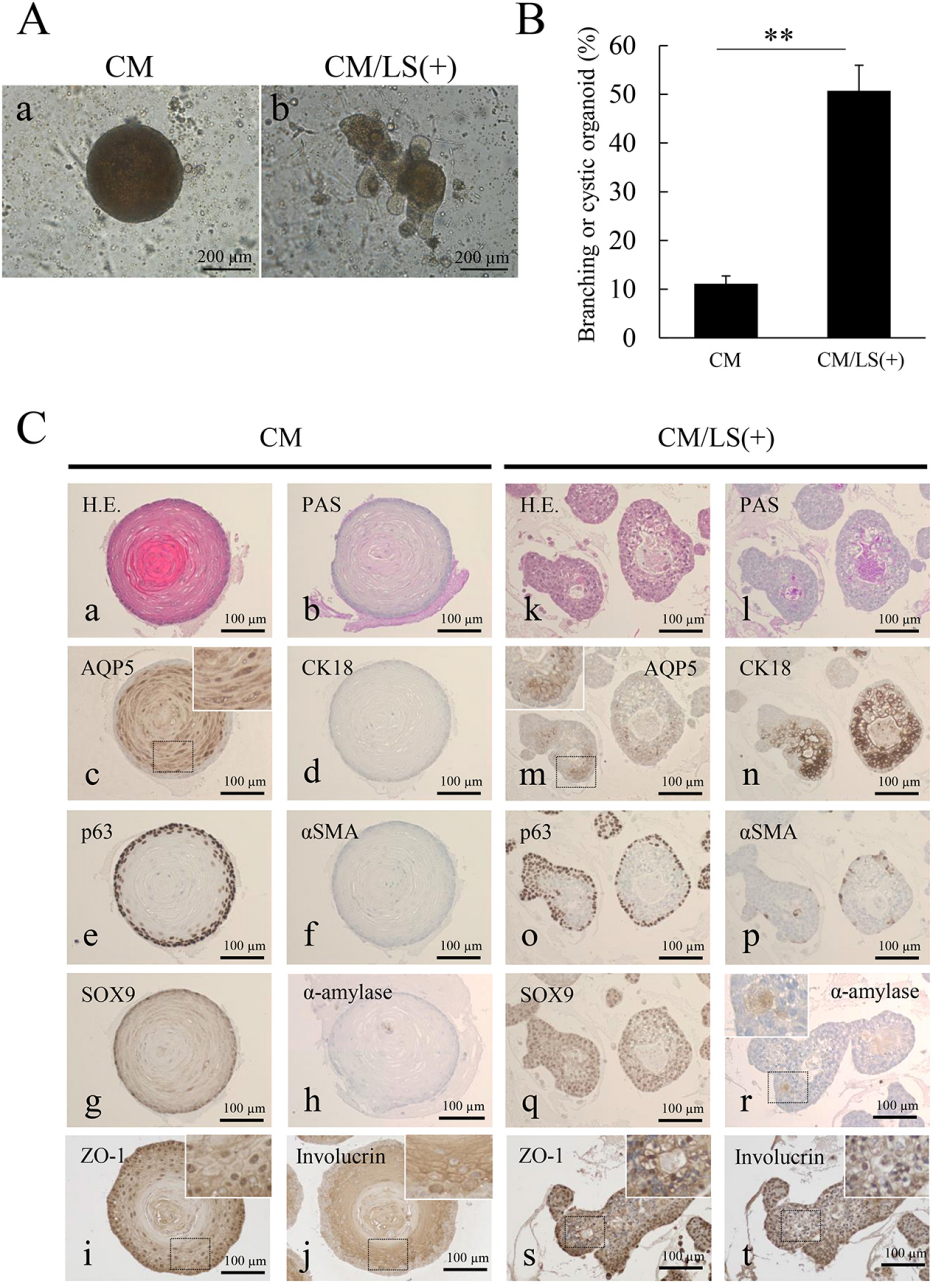
**The effect of BMP signaling inhibitors on human salivary-gland-derived organoids.** (A) Salivary gland organoids cultured in complete medium (CM; a) and in CM with LDN193189 (Alk2/3/6 inhibitor) and SB431542 (Alk4/5/7 inhibitor) [CM/LS(+); b] at day 16. Images show submandibular-gland-derived organoids. Scale bars: 200 µm. (B) Bar graph showing the percentage of organoids forming salivary gland-like branching or cystic morphologies in CM and CM/LS(+) culture conditions (*n*=3, >50 organoids). Data are from submandibular gland derived organoids at day 16 and are presented as mean+s.e.m. **P<0.01 (two-tailed Student's *t*-test). (C) Hematoxylin-Eosin staining (H.E.; a,k), Periodic Acid-Schiff staining (PAS; b,l) and immunohistochemical staining of aquaporin 5 (AQP5; c,m), keratin 18 (CK18; d,n), p63 (e,o), α-smooth muscle actin (α-SMA; f,p), SOX9 (g,q), α-amylase (h,r), zonula occludens protein 1 (ZO-1; i,s) and involucrin (j,t) in CM (a-j) or CM/LS(+) (k-t) culture conditions. Magnified images are shown in insets. Images show submandibular gland derived organoids at day 16. Scale bars: 100 µm.

Organoids cultured in CM revealed only round spheroid formation without any budding and/or branching features (Fig. 2A, panel a), but organoids cultured in CM/LS(+) showed remarkable morphological changes, including budding and/or branching (Fig. 2A, panel b). The percentage of salivary-gland-like organoids showing budding and/or branching morphology was significantly increased in CM/LS(+) compared to those in CM (Fig. 2B). The organoids cultured in CM showed a central keratinization, suggesting squamous metaplasia (Fig. 2C, panel a), and a peripheral lining of basal cells (Fig. 2C, panels a and e). Conversely, organoids cultured in CM/LS(+) showed remarkable budding and/or branching features with central hollow spaces reminiscent of human salivary glands (Fig. 2C, panel k). Periodic Acid-Schiff (PAS)-positive staining was observed in the central luminal spaces and the cells of organoids showing salivary-gland-like features in CM/LS(+), suggesting mucus production (Fig. 2C, panel l). However, PAS-positive staining was not detected in any parts of organoids cultured in CM (Fig. 2C, panel b). In addition, we analyzed expression patterns of several human salivary gland markers in the organoids. Immunohistochemically, organoids showing squamoid features cultured in CM were positive for AQP5 in the cytoplasm of inner cells (Fig. 2C, panel c and inset), p63 (also known as TP63) in the nuclei of peripheral basal cells (Fig. 2C, panel e), ZO-1 (TJP1; a marker of tight junctions) in the cell membrane (Fig. 2C, panel i and inset) and involucrin (a famous marker of keratinization) in the cytoplasm of inner squamous metaplastic cells (Fig. 2C, panel j and inset) but were negative for CK18 (Fig. 2C, panel d), α-SMA (Fig. 2C, panel f) and α-amylase (Fig. 2C, panel h). The peripheral basal cells were weakly positive for SOX9 (Fig. 2C, panel g). AQP5 is a famous marker of acini and is expressed in the apical membrane. CK18 is a salivary-gland-specific keratin and is expressed in both ducts and acini. α-SMA is a marker of myoepithelial cells. In contrast to these results, organoids showing salivary-gland-like features cultured in CM/LS (+) showed several different expression patterns of salivary gland markers. AQP5 was positive in the membrane of the inner cells (Fig. 2C, panel m and inset) and CK18 was strongly positive in the luminal inner cells (Fig. 2C, panel n). ZO-1 was also strongly positive in the membrane of inner cells (Fig. 2C, panel s and inset). The nuclei of both inner and peripheral cells showed positive staining for SOX9, known as a salivary gland cell marker (Chatzeli et al., 2017) (Fig. 2C, panel q). The nuclei of peripheral basal cells were also p63-positive (Fig. 2C, panel o). α-SMA staining was focally positive in some peripheral cells (Fig. 2C, panel p). Involucrin staining was negative in the cytoplasm of inner cells (Fig. 2C, panel t and inset). Furthermore, α-amylase was partly positive in the cytoplasm of the inner cells (Fig. 2C, panel r and inset). From these immunohistochemical findings, organoids cultured in CM/LS(+) showed some overlap of acinar and ductal markers, as well as limited myoepithelial differentiation. These findings suggested that inhibition of Alk signaling was necessary for the formation of salivary-gland-like organoids and that Alk signaling might play a role in the squamoid changes.

### Salivary gland organoid swelling assays

**Fig. 3.**
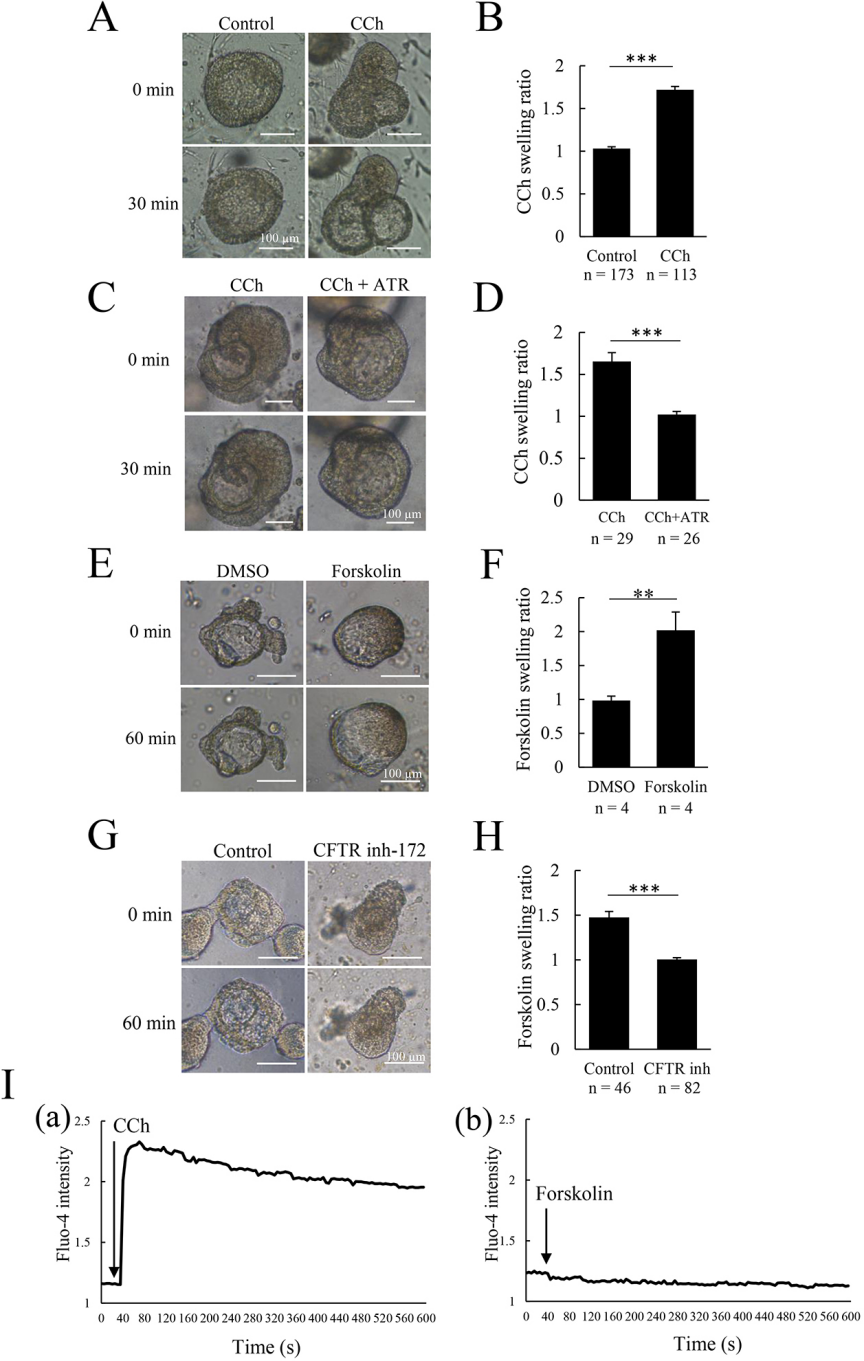
**Quantification of human salivary-gland-derived organoid swelling.** (A) Representative images of human salivary-gland-derived organoids either stimulated with carbachol (CCh) or in control conditions at 0 min and 30 min after stimulation. Images show labial-gland-derived organoids at day 9. (B) The swelling ratio of the normalized volume of organoids in control and CCh-stimulated conditions at 30 min after CCh stimulation (*n*=numbers of organoids). Data are from labial-gland-derived organoids at day 9. (C) Representative images of salivary gland organoids stimulated with CCh with or without atropine (ATR) at 0 min and 30 min after stimulation. Images show labial-gland-derived organoids at day 8. (D) The swelling ratio of the normalized volume of organoids at 30 min after CCh stimulation with or without atropine (*n*=numbers of organoids). Data are from labial-gland-derived organoids at day 8. (E) Representative images of salivary gland organoids stimulated with forskolin or DMSO at 0 min and 60 min after stimulation. Images show labial-gland-derived organoids at day 7. (F) The swelling ratio of the normalized volume of organoids after 60 min of DMSO or forskolin stimulation (*n*=numbers of organoids). Data are from labial-gland-derived organoids at day 7. (G) Representative images of salivary gland organoids stimulated with forskolin with or without CFTR inhibitor (CFTR inh-172) at 0 min and 60 min after stimulation. Images show labial-gland-derived organoids at day 10. (H) The swelling ratio of the normalized volume of organoids in control and CFTR-inhibited conditions at 60 min after forskolin stimulation (*n*=numbers of organoids). Data are from labial-gland-derived organoids at day 10. (I) Timecourse of Fluo-4 intensity in organoids stimulated with CCh (a) or forskolin (b). Data are from labial-gland-derived organoids at day 8. These experiments were replicated three times with similar results. Bar graphs show mean+s.e.m. **P<0.01, ***P<0.001 (two-tailed Student's *t*-test). Scale bars: 100 µm.

To evaluate whether our organoid system could be applied as an experimental system for the analysis of salivary gland diseases or disorders, we established a swelling model by using two different methods. First, we induced swelling of the organoids by stimulation with carbachol (CCh), a non-selective cholinergic agonist, because saliva secretion is well known to be induced by cholinergic stimulation (Larsson et al., 1989). Following CCh treatment, we observed organoid swelling immediately, whereas untreated control organoids were unaffected (Fig. 3A,B; Fig. S1A). The CCh-induced swelling was completely inhibited by pretreatment with atropine, which is a muscarinic receptor antagonist (Fig. 3C,D). Conversely, activation of the β-adrenergic receptor-mediated pathway using isoproterenol stimulation did not affect organoid swelling (Fig. S2A,B). Next, organoid swelling was induced by the activation of cystic fibrosis transmembrane conductance regulator (CFTR) using forskolin. CFTR is known as a cyclic AMP (cAMP)-activated anion channel and a member of the ATP-binding-cassette transporter family, and is expressed in the epithelial cells of several kinds of organs, including lung, intestine, pancreas and salivary gland. In these epithelial cells, CFTR plays an important role in fluid homeostasis (Dekkers et al., 2013). In salivary glands, CFTR is generally known to be expressed on the apical membrane of both acinar and ductal cells, and contributes to saliva production (Kulaksiz et al., 2002; Ishibashi et al., 2008). Interestingly, in intestinal organoids, forskolin raises the amount of intracellular cAMP and activates CFTR, inducing organoid luminal swelling. The forskolin-induced intestinal organoid swelling has been regarded as *in vitro* model mimicking *in vivo* cystic fibrosis in the intestinal epithelium (Dekkers et al., 2013, 2016). For these reasons, we applied forskolin to induce swelling in our established salivary gland organoids via the activation of CFTR. After the treatment with forskolin, we observed organoid swelling immediately, whereas DMSO-treated control organoids were unaffected (Fig. 3E,F; Fig. S1B). Finally, we examined the contribution of CFTR to the induction of organoid swelling by adding a CFTR chemical inhibitor, CFTR inh-172, in CM/LS(+). The inhibitor completely inhibited forskolin-induced organoid swelling (Fig. 3G,H). Furthermore, we investigated intracellular calcium levels in the organoids following CCh and forskolin stimulation (Fig. 3I). An increase in intracellular calcium was observed in organoids treated with CCh (Fig. 3I, panel a; Movie 1), and was not observed following forskolin stimulation (Fig. 3I, panel b). These data demonstrated that the swelling induced in the salivary gland organoids was CCh and CFTR dependent, and that the organoid swelling might be useful as an *in vitro* model for the analysis of salivary gland dysfunctions.

### TNF-α inhibited organoid swelling

**Fig. 4.**
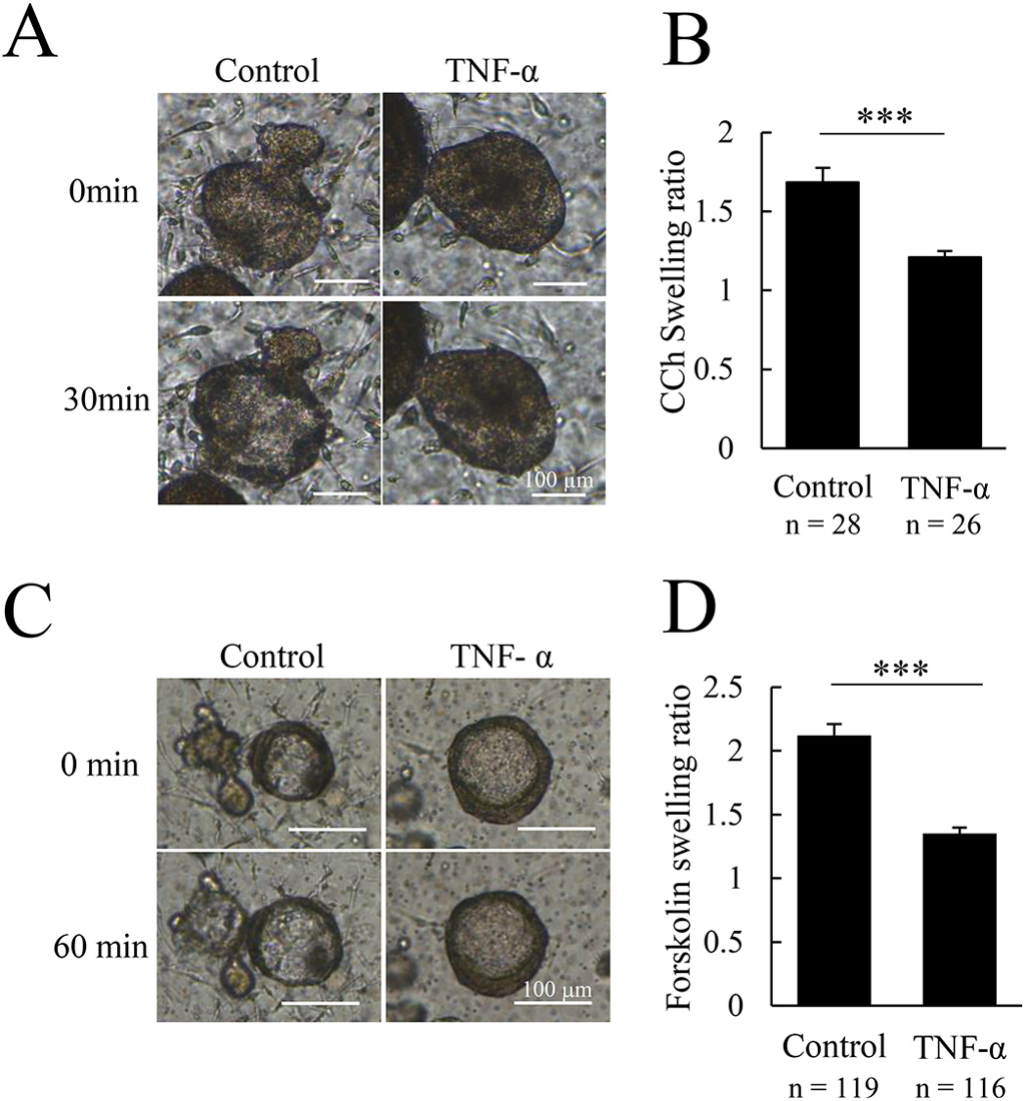
**TNF-α inhibits swelling of human salivary-gland-derived organoids.** (A) Images of TNF-α untreated (control) and treated human salivary-gland-derived organoids stimulated with carbachol (CCh). Organoids were treated with 100 ng/ml TNF-α for 72 h before CCh-induced swelling. Representative images at 0 min and 30 min after stimulation are shown. Images show labial-gland-derived organoids at day 14. (B) The swelling ratio of the normalized volume of organoids in control and TNF-α pretreated conditions at 30 min after CCh stimulation (*n*=numbers of organoids). Data are from labial-gland-derived organoids at day 14. (C) Images of TNF-α untreated (control) and treated human salivary-gland-derived organoids stimulated with forskolin. Organoids were treated with 100 ng/ml TNF-α for 72 h before forskolin-induced swelling. Representative images at 0 min and 60 min after stimulation are shown. Images show labial-gland-derived organoids at day 7. (D) The swelling ratio of the normalized volume of organoids in control and TNF-α pretreated conditions at 60 min after forskolin stimulation (*n*=numbers of organoids). Data are from labial-gland-derived organoids at day 7. Bar graphs show mean+ s.e.m. ***P<0.001 (two-tailed Student's *t*-test). Scale bars: 100 µm.

**Fig. 5.**
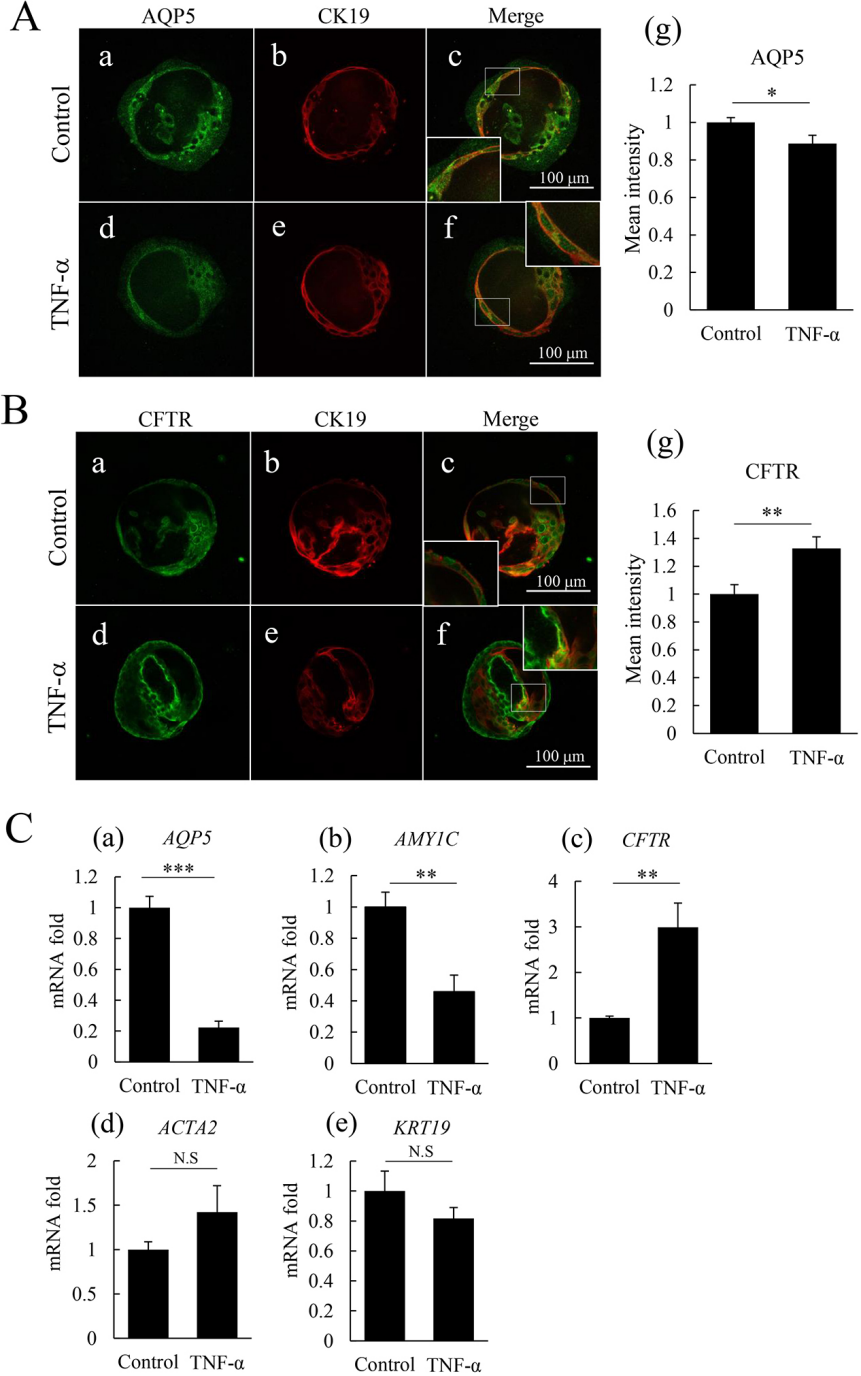
**TNF-α inhibits expression of aquaporin 5 in human salivary-gland-derived organoids.** (A) Immunofluorescence staining of aquaporin 5 (AQP5, green) and keratin 19 (CK19, red) in human salivary-gland-derived organoids with or without (control) 100 ng/ml TNF-α treatment for 72 h. Aquaporin 5 expression is repressed by TNF-α treatment (d,f). Merged images are shown as panels c and f. Magnified images are shown in insets. Scale bars: 100 µm. The bar graph indicates the intensity of AQP5-positive staining (g) (*n*=5). Images show labial-gland-derived organoids at day 7. (B) Immunofluorescence staining of cystic fibrosis transmembrane conductance regulator (CFTR, green) and keratin 19 (CK19, red) in human salivary-gland-derived organoids with or without (control) 100 ng/ml TNF-α treatment for 72 h. CFTR expression is upregulated by TNF-α treatment (d). Merged images are shown in panels c and f. Magnified images are shown in insets. Scale bars: 100 µm. The bar graph indicates the intensity of CFTR positive staining (g) (*n*=5). Images show labial-gland-derived organoids at day 7. (C) mRNA expression in organoids treated with 100 ng/ml TNF-α for 48 h. Real-time qPCR analyses show a statistically significant decrease in *AQP5* (a) and α-amylase (*AMY1C*; b) expression and a significant increase in *CFTR* expression in organoids treated with TNF-α (c). No significant change is seen in α-smooth muscle actin (*ACTA2*; d) and keratin 19 (*KRT19*; f) mRNA expression (*n*=4 experiments). Data are from parotid-gland-derived organoids at day 9. Bar graphs show mean+s.e.m. *P<0.05; **P<0.01; ***P<0.001; N.S., not significant (two-tailed Student's *t*-test).

Sialadenitis is one of the main factors of hyposalivation. In this inflammatory condition, it is well known that TNF-α is upregulated and plays some pivotal roles (Limaye et al., 2019; Kang et al., 2011; Hong et al., 2019). We therefore used our organoid system to analyze the mechanisms of this inflammatory condition as a disease model. In this experiment, we treated organoids with TNF-α to mimic the inflammatory condition in human salivary glands recognized in sialadenitis and analyzed the pathophysiological changes using the CCh- and forskolin-induced swelling assays. First, TNF-α was added 3 h before starting the organoid swelling assays, and we investigated the short-term effect of TNF-α stimulation. In this condition, no significant changes in swelling were seen (data not shown). Next, we tested the effect of long-term TNF-α stimulation and observed that organoid swelling was significantly suppressed (Fig. 4A-D). When we applied interferon-γ (IFN-γ), another inflammatory cytokine, organoid swelling was not affected (Fig. S2C,D). During fluid transport, CFTR activation causes Cl^−^ secretion to the lumen from the epithelia and induces fluid transport to the lumen from the epithelia. AQP5, a water channel, is one possible route by which water moves to the lumen from the epithelia. In our study, immunofluorescence revealed that AQP5 expression was downregulated in luminal cells of TNF-α-treated organoids (Fig. 5A, panels a,d,g; Fig. S2E). Reverse transcription qPCR (RT-qPCR) analyses also showed that there was a significant decrease in the expression levels of *AQP5* mRNA in organoids following TNF-α treatment (Fig. 5C, panel a). In this condition, *AMY1C* (α-amylase mRNA) was also significantly decreased (Fig. 5C, panel b). Interestingly, in the TNF-α-treated condition, CFTR protein expression was conversely upregulated in luminal cells, as determined by immunofluorescence staining analyses (Fig. 5B, panels a,d,g; Fig. S2F) and the expression levels of *CFTR* mRNA were significantly upregulated, as quantified in RT-qPCR analyses (Fig. 5C, panel c). Nevertheless, mRNA levels of other salivary gland markers, *KRT19* (keratin 19) and *ACTA2* (α-SMA) showed no significant change (Fig. 5C, panels d and e). These results revealed that TNF-α suppressed organoid swelling by decreasing AQP5 expression. Thus, the salivary gland organoid that we newly established is a possible evaluation model to quantitate the effects of TNF-α in inflammatory conditions affecting the salivary glands.

## DISCUSSION

Organoid experimental models are useful for studying the processes of tissue and organ development, tissue repair and regeneration following damage, and carcinogenesis, as well as having applications in drug discovery. In this study we established a novel system for producing human salivary-gland-derived organoids, which we then applied to an inflammatory disease model.

In 2D monolayer cultures, human primary acinar cells lose biological functions, including acinar-specific protein expression, granule formation, calcium mobilization, trans-epithelial resistance and polarized amylase secretion after β-adrenergic receptor stimulation (Lombaert et al., 2017). On the other hand, in 3D gel matrix culture, human salivary gland cells show salivary gland protein expression, polarization and expansion in long-term cultures (Feng et al., 2009; Pringle et al., 2016; Hosseini et al., 2018). In our 3D culture system, organoids could be expanded for at least 1 month and could maintain their own functions, such as CCh- and CFTR-induced swelling.

To optimize organoid culture conditions, we used serum-free medium with and without some cytokines. Some previous reports described that cytokines such as Wnt3a, R-spondin1, FGF7 and FGF10 are important for organogenesis, development and regeneration of salivary glands (Matsumoto et al., 2016; Chatzeli et al., 2017). Thus, we applied these cytokines in our culture conditions. In addition, to suppress squamoid differentiation, we used inhibitors of Alk signaling (also known as TGF-β/Smad signaling), LDN193189 and SB431542. These inhibitors have been used in the formation of organoids from pluripotent stem cells. Both these inhibitors play essential roles in inducing non-neural ectoderm, including oral ectoderm, from mouse embryonic stem cells. In particular, LDN193189 inhibits the differentiation from non-neural ectoderm into epidermis in salivary gland differentiation (Tanaka et al., 2018). Initially, squamous differentiation with marked central keratinization, but no acinar cell differentiation, was seen in organoid spheroids cultured in CM, which contained neither inhibitor. In sialadenitis, squamous metaplasia of ductal epithelial cells and loss of acini are often seen (Marshak et al., 1987). From these findings, the culture conditions in CM without Alk signaling inhibitors might be considered a part of an inflammatory condition similar to that occurring in sialadenitis. It is well known that TGF-β, a famous pro-inflammatory cytokine, is elevated in such inflammatory conditions found in salivary gland fibrosis (Hall et al., 2010). Interestingly, a previous study described that overexpression of TGF-β inhibits mouse salivary gland development and specific functions (Hall et al., 2010). In addition, it was also reported that TGF-β plays some important roles in squamous metaplasia in human airway cells (Tanabe et al., 2012). Thus, TGF-β might be a critical factor for squamous metaplasia and loss of acini during the formation of salivary gland organoids, and, conversely, inhibition of this pathway might be important for maintaining the organoid structures and functions.

For the use of human salivary gland organoids as a model to evaluate secretory functions, we used a forskolin-induced organoid swelling system. This system was initially established in intestinal organoids to evaluate CFTR functions in patients with cystic fibrosis (Dekkers et al., 2013). CFTR is expressed not only in intestine but also in salivary glands, on the apical cell membrane in both acinar and ductal cells, and contributes to saliva production (Kulaksiz et al., 2002; Ishibashi et al., 2008). In these experimental systems, forskolin upregulates intracellular cAMP, and activates CFTR. CFTR activation has been known to be involved in Cl^−^ secretion into the extracellular lumen and to induce fluid transport via AQP5. From these points of view, analyses using organoid swelling models might be a useful system for the evaluation of salivary gland secretory function.

It is well known that TNF-α expression is elevated and plays important roles in salivary gland diseases such as Sjögren syndrome and IgG4-related sialadenitis (Kang et al., 2011; Hong et al., 2019). Intriguingly, one previous study described that TNF-α induces suppression of AQP5 expression in immortalized normal human salivary gland acinar cells, and this is due to epigenetic regulation through the suppression of histone H4 acetylation (Yamamura et al., 2012). In our organoid experiments, TNF-α did not affect the organoid swelling during a short timecourse. However, long-term TNF-α stimulation suppressed *AQP5* mRNA and protein expression. Thus, alteration of mRNA levels through epigenetic mechanisms of regulation might occur in our organoids. On the other hand, *CFTR* mRNA and protein expression was upregulated upon long-term (72 h) TNF-α stimulation. Further investigations are needed for the clarification of this alternative expression of AQP5 and CFTR upon TNF-α stimulation. In this study, we focused on fluid transport via AQP5, and have not mentioned protein secretion. Further investigation is needed to detect protein secretion in organoids to confirm whether the components of secretory products in organoids are similar to those of the saliva secreted from human salivary glands.

In summary, we have newly established a system to produce human salivary-gland-derived organoids and showed a useful assay system using these organoids as an evaluation model to quantitate the effects of TNF-α on an inflammatory condition. Our newly established organoids and their culture systems could become useful tools, not only in the analysis of morphological and functional changes associated with salivary gland dysfunctions, but also in the establishment of disease models, drug screening systems and regenerative study models of damaged salivary glands.

## MATERIALS AND METHODS

### Salivary gland isolation and organoid culture

This clinical study using patients' information and materials was performed under the permission of the ethics committee in Fukuoka Dental College (No. 406). Informed consent was obtained for all tissue donors and all clinical investigations were conducted according to the principles expressed in the Declaration of Helsinki. Salivary glands were obtained from patients with mucous cyst and head and neck tumors who underwent operations at Fukuoka Dental College hospital. Eighteen patients agreed to donation of salivary glands. Four submandibular glands, two sublingual glands, three parotid glands, one palatine gland and nine labial glands were used for the procedure of each organoid formation. Resected salivary glands were washed in cold PBS, chopped into ∼1-mm pieces, then digested with 0.63 mg/ml collagenase type II (Worthington Biochemical Corporation, Lakewood, NJ, USA) and 0.5 mg/ml hyaluronidase (Sigma-Aldrich, St Louis, MO, USA) in Minimum Essential Media (MEM) (Sigma-Aldrich) at 37°C for 2 h. After filtering through 70-µm and 40-µm cell strainers and washing in PBS, 5×10^3^ cells (single cells or aggregates of 2-5 cells) were suspended in 50 μl of the liquid sol phase of Matrigel™-growth factor reduced (GFR) (BD Bioscience, Bedford, MA, USA) at 4°C, and were seeded in 24-well tissue culture plates. Cells in the solid gel phase of Matrigel™-GFR were cultured by being overlaid with 500 μl of complete medium (CM) at 37°C in 5% CO_2_ for each objective period [CM consists of DMEM/F12 medium (Sigma-Aldrich) containing Pen/Strep antibiotics (Sigma-Aldrich), Glutamax (Thermo Fisher Scientific, Waltham, MA, USA), N2 (Thermo Fisher Scientific), 20 ng/ml EGF (Sigma-Aldrich), 100 ng/ml FGF7 (Wako, Osaka, Japan), 100 ng/ml FGF10 (PeproTech, Rocky Hill, NJ, USA), 0.05 mg/ml heparin (Sigma-Aldrich), 100 ng/ml Wnt3a (R&D Systems, Minneapolis, MN, USA), 500 ng/ml R-spondin1 (PeproTech), Insulin-Transferrin-Selenium (Thermo Fisher Scientific), 10 µM Y-27,632 (Wako) and 50 nmol/l hydrocortisone (Tokyo kasei, Tokyo, Japan)]. To confirm the effect of Alk inhibitors (LDN193189 and SB431542), which were used in a process to form salivary glands from mouse pluripotent stem cells in a previous report (Tanaka et al., 2018), on the induction of human salivary-gland-derived organoids in our culture system, 100 nM LDN193189 (L; Sigma-Aldrich) and 1 µM SB431542 (S; Abcam, Cambridge, UK) were added to the CM throughout the culture period [CM/LS(+)]. The culture medium was changed every 2-3 days.

### Scanning electron microscopy

After fixation with 4% paraformaldehyde (PFA) for 1 h and postfixation with 1% osmium tetroxide solution for 1 h, organoids were dehydrated in ethanol and critical point dried in liquid CO_2_, then sputter coated with platinum (Pt). Samples were investigated using a JEM-6330F scanning electron microscope (JEOL, Tokyo, Japan).

### Immunostaining of organoids

4% PFA-fixed and paraffin-embedded organoid tissue blocks were cut into 4-μm-thick sections for H.E., Periodic Acid-Schiff (PAS) and immunohistochemical staining. Antigen retrieval was performed for all sections by an autoclave treatment at 121°C for 5 min in 0.01 M citrate buffer, pH 6.0. Immunostaining was performed using an EnVision/horseradish peroxidase (HRP) kit (DAKO-Agilent Technologies Co., Santa Clara, CA, USA). Briefly, the sections were treated with a 0.1% hydrogen peroxide-methanol solution to inhibit endogenous peroxidase activity and with 5% BSA in Tris-buffered saline (TBS) to block any non-specific binding of primary antibodies. Subsequently, each section was incubated with the primary antibody against AQP5 (1:500 dilution; #AQP-005, Alomone labs, Jerusalem, Israel), CK18 (1:100; clone DC 10, DAKO), p63 (1:100; clone 4A4, DAKO), α-smooth muscle actin (1:100; clone 1A4, DAKO), CK19 (1:100; clone A-3, Santa Cruz Biotechnology, Santa Cruz, CA, USA), α-amylase (1:200; clone G-10, Santa Cruz Biotechnology), SOX9 (1:100; #AB5535, Millipore, Burlington, MA, USA), ZO-1 (1:100; #21773-1-AP, Proteintech, Rosemont, IL, USA) and involucrin (1:100; SY5, Santa Cruz Biotechnology) at 4°C overnight. After washing in TBS, these sections were then incubated with HRP-conjugated anti-rabbit or anti-mouse secondary antibody. The peroxidase activity was visualized using 0.1% 3,3′-diaminobenzidine and 0.01% hydrogen peroxide in TBS. For immunofluorescence staining, after incubation with each primary antibody, the section was incubated with Alexa Fluor 594-conjugated goat anti-rabbit IgG (1:1000 dilution; #A32740, Thermo Fisher Scientific) or Alexa Fluor 488-conjugated goat anti-mouse IgG (1:1000 dilution; #A32723, Thermo Fisher Scientific) secondary antibody. Then, sections were mounted using VECTASHIELD with DAPI (Vector Lab., Burlingame, CA, USA). Micrographs were visualized and captured at the appropriate wavelength using an LSM 710 fluorescence laser microscope (Carl Zeiss Inc., Oberkochen, Germany). The images of H.E. and immunohistochemical staining were captured using an AXIO Vert.A1 microscope (Carl Zeiss Inc). The images were processed using the ZEN 2010B Sp1 Ver. 6.0.0.485 software (Carl Zeiss Inc.). Intensity of fluorescence was quantified using ImageJ (Schneider et al., 2012).

### Organoid swelling assays

Organoids (culture day 7 to 9) were aliquoted at 40-70 organoids per well in a 24-well plate and cultured in Matrigel covered with CM/LS(+). In the swelling assays, 100 µM carbamoylcholine chloride (carbachol; CCh; Sigma-Aldrich) and 5 µM forskolin (Tokyo kasei) were added. For the muscarinic M1 blocking, 100 nM atropine (Tokyo kasei) was added 30 min before CCh treatment. For CFTR inhibition, organoids were preincubated in CM/LS(+) with 50 µM CFTR inh-172 (Sigma-Aldrich) for 3 h. For tumor necrosis factor-α (TNF-α) stimulation, organoids were preincubated in CM/LS(+) with 100 ng/ml TNF-α (Sigma-Aldrich) for 72 h. Morphological changes of organoids were analyzed by BZ-X710 live-cell microscopy (KEYENCE, Tokyo, Japan). 3-5 wells were used for each inhibitory or stimulatory condition group in each analysis assay. The swelling ratio between each experimental condition and control group was calculated by comparing the mean value for the experimental condition group with the mean value of the control group. The volume of each organoid (*V*) was quantified using ImageJ (Schneider et al., 2012). *V*=4/3π *r*^3^ (*r*, radius; *r* was measured using Image J).

### Calcium imaging

Calcium imaging analysis was performed using a Calcium Kit-Fluo 4 (Dojindo, Kumamoto, Japan), according to the manufacturer's protocol. In brief, organoids were incubated with the Fluo-4 solution for 60 min at 37°C. After washing, the fluorescence was captured via image acquisition using BZ9000 live-cell microscopy (KEYENCE), followed by treatment of the organoids with 100 µM of carbachol or 5 µM of forskolin.

### RNA isolation and RT-qPCR

Organoids were isolated from Matrigel™-GFR using Cell Recovery Solution (Corning, Bedford, MA, USA). Total RNA isolation was performed using the ReliaPrep RNA Cell Miniprep System (Promega, Madison, WS, USA), and cDNA synthesis was conducted using the ReverTra Ace (Toyobo, Osaka, Japan), according to the manufacturer's protocol. RT-qPCR was performed using Fast start essential DNA green master (Roche, Basel, Switzerland). The samples were analyzed, and message levels of *CFTR* or *AQP5* were normalized to the corresponding *GAPDH* expression level. Primers used for RT-qPCR were as follows: *CFTR* forward, 5′-TTGGATGACCTTCTGCCTCT-3′; *CFTR* reverse, 5′-CTCCTGCCTTCAGATTCCAG-3′; *AQP5* forward, 5′-GCTCACTGGGTTTTCTGGGTA-3′; *AQP5* reverse, 5′-TCCATGGTCTTCTTCCGCTC-3′; *KRT19* forward, 5′-TGAGGAGGAAATCAGTACGCT-3′; *KRT19* reverse, 5′-CGACCTCCCGGTTCAATTCT-3′; *ACTA2* forward, 5′-GACTTCCGCTTCAATTCC-3′; *ACTA2* reverse, 5′-GTTAGGACCTTCCCTCAG-3′; *AMY1C* forward, 5′-AATTGATCTGGGTGGTGAGC-3′; *AMY1C* reverse, 5′-CTTATTTGGCGCCATCGATG-3′; *GAPDH* forward, 5′-ATCACCATCTTCCAGGAGCGAG-3′; and *GAPDH* reverse, 5′-TGGCATGGACTGTGGTCATG-3′.

### Statistical analyses

All data were expressed as the mean±s.e.m. A two-tailed Student's *t*-test was applied for the comparison between two groups. Statistical significance was set as *P<0.05, **P<0.01 and ***P<0.001.

## Supplementary Material

Supplementary information
