# Supplementary material for: Inhibition of Alk signaling promotes the induction of human salivary-gland-derived organoids
Source: Dis Model Mech. 2020 Sep 28;13(9):dmm045054. doi: 10.1242/dmm.045054 (PMC7541338; doi:10.1242/dmm.045054)
Supplement: Supplementary information [file dmm-13-045054-s1.pdf]

Supplementary information

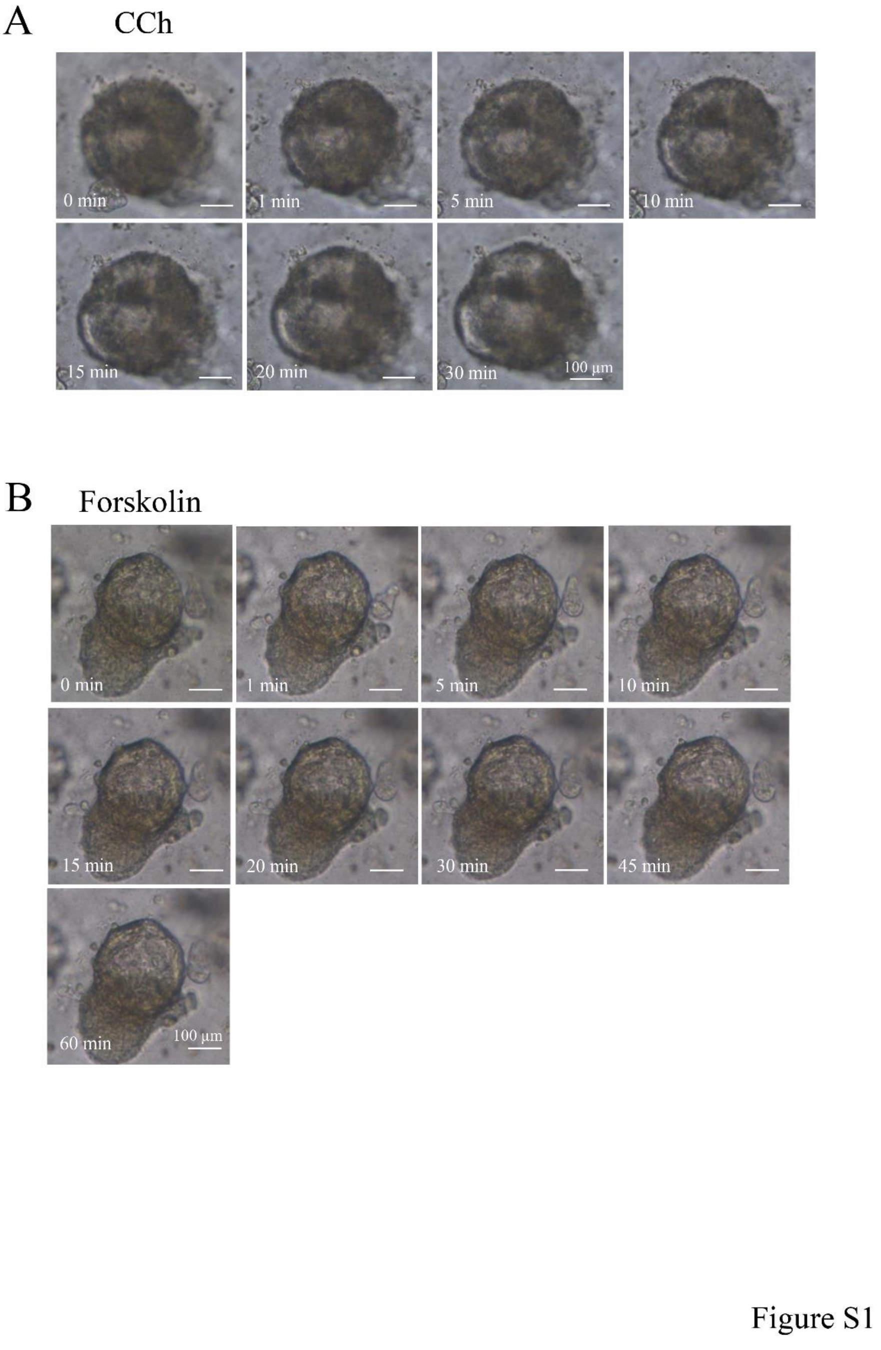

Figure S1.

(A) Time lapse imaging of the organoid swelling treated with 100 μM carbachol. (B) Time lapse imaging of the organoid swelling treated with 5 μM forskolin. Labial gland derived organoids, day 8. Scale bar: 100 μm.

Disease Models & Mechanisms • Supplementary information

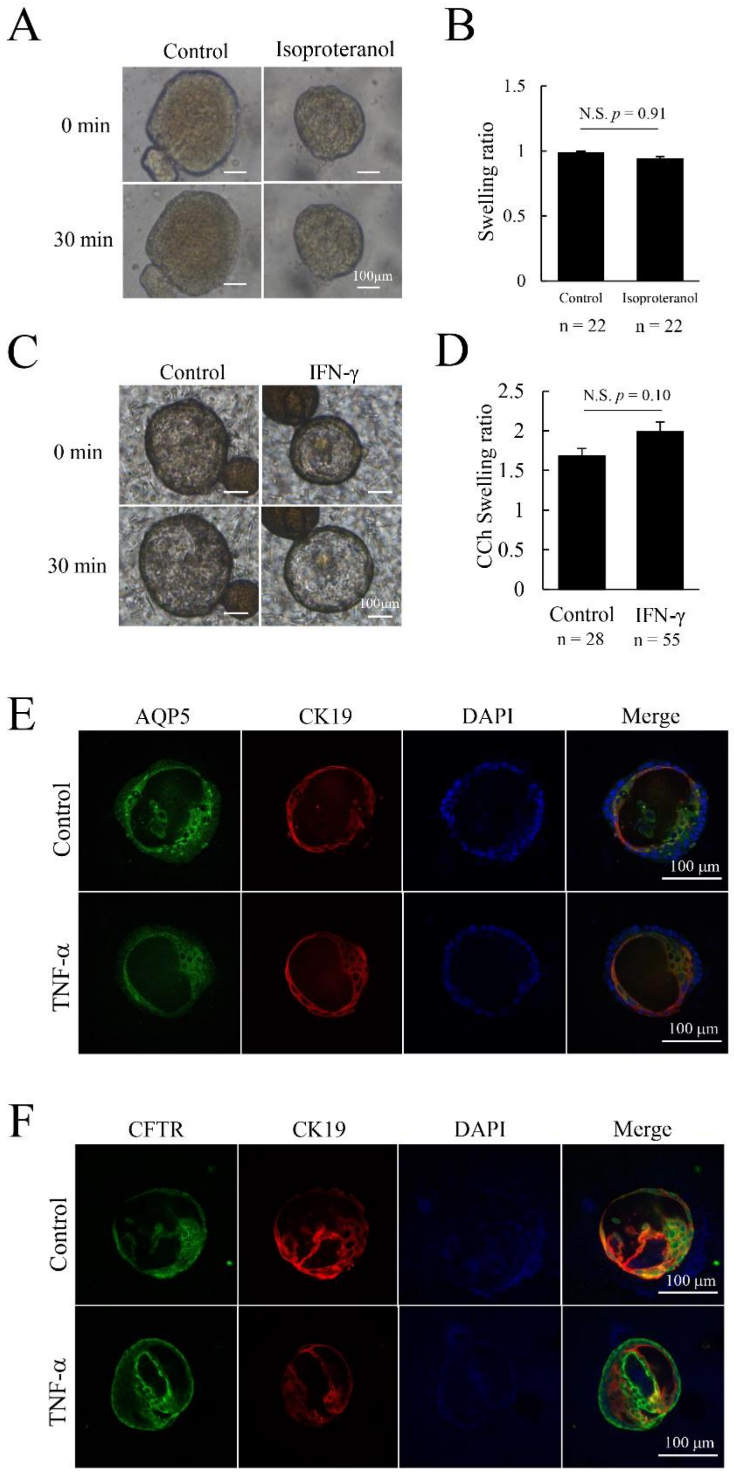

Figure S2

Figure S2.

(A) Microscopic images of salivary gland organoids stimulated with 10 μM isoproterenol (Sigma-

Disease Models & Mechanisms • Supplementary information

Aldrich) or DMSO. Representative images at 0 min and 30 min time points after stimulation are shown. Labial gland derived organoids, day 8. Scale bar: 100 μm. (B) The bar graph indicates the comparison of the swelling ratio of the normalized volume of organoids between the DMSO and isoproterenol stimulated state at 30 min after the stimulation (n = numbers of organoids). Labial gland derived organoids, day 8. (C) Microscopic images of IFN-γ untreated (control) and treated human salivary gland-derived organoids stimulated with carbachol (CCh). Organoids were treated with 10 ng/ml IFN-γ (PeproTech) for 72 h before CCh-induced swelling. Representative images at 0 min and 30 min time points after stimulation are shown. Labial gland derived organoids, day 14. Scale bar: 100 μm. (D) The bar graph indicates the comparison of the swelling ratio of the normalized volume of organoids between the control and CCh stimulated state at 30 min after CCh stimulation (n = numbers of organoids). Labial gland derived organoids, day 14. (E) Immunofluorescent cytochemical staining of aquaporin 5 (AQP5: green), keratin 19 (CK19: red) and nuclear staining (DAPI: blue) in organoids with or without (control) 100 ng/ml TNF-α treatment for 72 h. Merged images are shown in the right panels. Labial gland derived organoids, day 7. Scale bar: 100 μm. (F) Immunofluorescent cytochemical staining of cystic fibrosis transmembrane conductance regulator (CFTR: green), keratin 19 (CK19: red) and nuclear staining (DAPI: blue) in organoids with or without 100 ng/ml TNF-α treatment for 72 h. Merged images are shown in the right panels. Labial gland derived organoids, day 7. Scale bar: 100 μm.

Disease Models & Mechanisms • Supplementary information

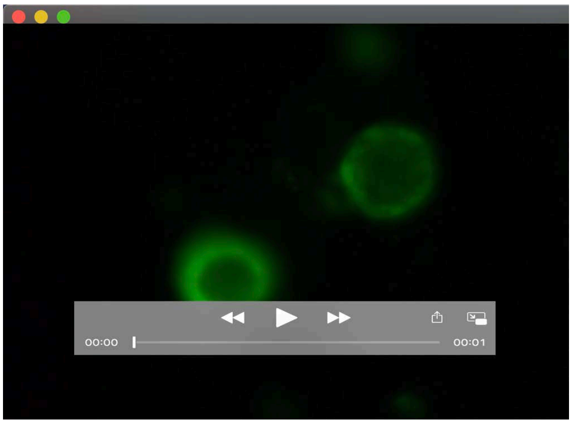

Movie 1

The movie shows a time lapse calcium imaging using Fluo-4. Organoids were treated with 100 µM carbachol, and recorded for 10 minutes. Green fluoresece shows calcium release in the organoid cells. Labial gland derived organoids, day 8.
